# Unraveling the adaptive chemical traits of *Rhamnidium elaeocarpum* Reissek in response to fire in pantanal wetlands

**DOI:** 10.1038/s41598-023-38725-2

**Published:** 2023-07-22

**Authors:** Camila Sório Siqueira, Vanessa Samúdio Dos Santos, Carlos Alexandre Carollo, Geraldo Alves Damasceno-Junior

**Affiliations:** 1 Programa de Pós-Graduação Em Biologia Vegetal, Universidade Federal de Mato Grosso do Sul, Campo Grande, Mato Grosso do Sul, Brazil; 2 Laboratório de Produtos Naturais e Espectrometria de Massas (LAPNEM), Faculdade Ciências Farmacêuticas, Alimentos e Nutrição (FACFAN), Universidade Federal de Mato Grosso do Sul, Campo Grande, Mato Grosso do Sul, Brazil; 3 Laboratório de Ecologia Vegetal, Instituto de Biociências (INBIO), Universidade Federal de Mato Grosso do Sul, Campo Grande, Mato Grosso do Sul, Brazil

**Keywords:** Chemical ecology, Metabolomics, Natural products

## Abstract

We conducted a study on the effects of fire on *Rhamnidium elaeocarpum*, a widely distributed woody species found in the Pantanal wetlands, using LC–MS metabolomics, total phenolic and tannin content analysis, and thermogravimetric behavior. We sampled individuals from four groups: No Fire, Fire 2019, Fire 2020, and APD 20 (individuals whose aerial parts had died during the 2020 fire event). We found that recent fires had no significant impact on the species' phenolic metabolism except for those in the fourth group. These specimens showed a decline in secondary metabolites due to leaching. The high levels of phenolics in *R. elaeocarpum* suggest that this species has a biochemical tolerance to the stress caused by seasonal fires. Metabolomic profiling revealed the presence of proanthocyanidin oligomers, which protect against oxidative stress and post-fire environmental disturbances. However, the passage of fire also led to a high incidence of toxic karwinaphthopyranone derivatives, which could be a concern for the species' medicinal use. Finally, the thermogravimetric analysis showed that the species is thermotolerant, with an intrinsic relationship between the secondary compounds and thermotolerance. Our research has deepened the comprehension of how fire affects the metabolic processes of woody plants. The challenge now lies in determining if the identified chemical changes are adaptive characteristics that evolved over time or merely transient responses to external environmental stimuli.

## Introduction

Global transformations have led to widespread wildfires, causing severe impacts on plant communities, society, and economy^1^. Even humid landscapes are not spared due to the prolonged dry periods^1,2^. The Brazilian Pantanal, the most extensive wetland in the world, has experienced an increase in fire incidence and severity^3^. In 2020, the Pantanal suffered the longest and most severe drought in the last 60 years^4^, which, combined with high temperatures and reduced soil moisture, led to the largest forest fire ever recorded in the area (Libonati et al. 2020). The National Institute for Space Research (INPE) and ALARMES alert system reported that about 3.9 million hectares of the biome were affected by fire in 2020, with more than 15,000 fires registered, three times more than the previous year^5–7^. Furthermore, it is essential to underscore the substantial impact of human activities in exacerbating fire events alongside climatic conditions^3,7,8^. The accumulation of dry biomass, resulting from factors such as drought and deforestation, coupled with the uncontrolled use of fire for land clearance purposes, significantly contributes to the escalation of fire incidents and the creation of favorable conditions for their rapid propagation^2,3^.

Fire significantly alters plant community composition and structure, favoring more fire-tolerant species and restricting vulnerable ones^9,10^. Specific physiological and anatomical adaptations such as bark thickening, rapid growth, protection of photosynthetic structures, and improved germination power have been linked to fire events^11^. However, the ability of plant communities to recover after a fire event is influenced by the frequency, intensity, and duration of fires^12^. This variability in ecological resilience to fire highlights the importance of adaptive traits in plants to a specific fire regime and the potential threat posed by changes in stress patterns^13–15^.

Considering the various strategies that plants develop to survive fire events, the biochemical mechanism is vital, although it is amongst the least elucidated in fire ecology. The chemical defense against biotic and abiotic stresses is also mediated by the secondary metabolism of plants^16^. Several metabolites are involved in this process, varying according to their physiological role^17^. For instance, phenolics are stress bioindicators of changes due to their abundance in specific environmental conditions^18,19^. Cannac et al.^20^ observed an increased phenolic biosynthesis in *Pinus laricio* in response to fires. The relationship between phenolic biosynthesis and fire was also reported in thermotolerant species in the Pantanal riparian forests in Brazil. Da Silva et al.^21^ recorded higher concentrations of total phenolic and tannin in resistant species compared with those thermosensitive. Additionally, phenolics, mainly tannins, have low thermal conductivity^22–24^, which could have important roles during a fire event.

In post-fire environments, tannin plays a vital defense mechanism against herbivores by forming a phenolic barrier^25^. In addition to providing mechanical resistance, reducing flammability, and preventing damage from microorganisms, tannins are the second most abundant class of phenolic compounds^26^. They are oligomeric products formed by the flavonoid biosynthetic pathway. They can be categorized into two classes: hydrolyzable tannins, hydrolyzable phenol/sugar ester linkages, and condensed tannins, catechin derivatives connected by C–C or C–O–C bonds. The molecular size is essential to their biological activities, the large number of hydroxyls in tannins' structure and their conjugated bonds make them potent antioxidant agents, protecting cells from free radical damage^27,28^.

Different plant species exhibit varying responses to environmental conditions, ultimately leading to the selection of the most suitable species for specific habitats. *Rhamnidium elaeocapum* Reissek, commonly known as *cabriteira* or *cabrito*, possesses functional characteristics that facilitate its rapid propagation and dominance in environments prone to frequent fires across diverse flood gradients^29,30^. Due to its significance as a food source for numerous bird species, it is highly recommended for mixed reforestation efforts to restore degraded areas^31^.

The Pantanal wetlands represent a suitable scenario to analyze the impact of fire events on the secondary metabolites. In addition to the need to expand knowledge about the relationship between fire and wetlands, since an increasing occurrence of extreme events is expected due to climate change and land use^1^. We hypothesize that secondary metabolites accumulate more in response to recent fire events, and the chemical profiles contribute to its thermotolerance. Therefore, in this study, we sought to: (a) evaluate the effect of a recent fire, over two years, on the accumulation of total phenolic compounds and tannins.; (b) verify if there is an accumulation of phenolic compounds in older plants; (c) discover the chemical response to fire over a time scale; (d) determine whether thermogravimetric properties change in individuals subjected to more recent fire events; (e) estimate the extent to which secondary metabolites influence the thermal stability of this species. In addition to expanding the insufficient chemical knowledge of the species, the study adds to the discussion about the effect of fire on ecophysiological behavior, contributing to the understanding of plant resistance and resilience mechanisms against environmental stresses.

## Methods and materials

The experimental research and field studies, including the collection of plant material, were conducted in accordance with institutional, national, and international guidelines and legislation. Collected samples were registered in the National System of Genetic Resource Management and Associated Traditional Knowledge (SisGen), under the registration code ABEE5E8. This mandatory registration process is an essential prerequisite for obtaining authorization to conduct research activities in Brazil.

### Study area and imagery

The study was conducted in *capões* forests located at *São Bento* Farm (19° 28′ 49.26'' S, 57° 00′ 57, 38'' W) in the Pantanal subregion Abobral, in the city of Corumbá, State of Mato Grosso do Sul, Brazil. The average annual rainfall is about 1070 mm, with a yearly mean temperature of 25.5 °C^32^. The climate is seasonal and characterized by a rainy period between October and March. The maximum point of the flooding regime occurs in April, when the flatlands fields are flooded^33,34^. Soils have a high base content due to the deposition of calcareous shells and their color darkens towards the center due to the gradual accumulation of organic matter^35^.

The study had a time-lapse of 21 years (1999–2020) on regional fire episodes. We used the information collected from 1999 to 2016^36^. In the subsequent years (2017–2020), we acquired images from the Landsat 8 OLI sensor with a spatial resolution of 30 m, specifically utilizing bands B3, B6, and B7. This composition was selected to emphasize the presence of fire through the combination of short-wave infrared bands. Data was downloaded from INPE's catalog^37^. Satellite imagery has documented the presence of intense fires in 2019 and 2020, as well as areas that remained fireless for a significant period. The most recent recorded fire events in these non-burning areas date back to 2009.

### Selected specie and sampling

We chose *R. elaeocarpum*, as a model, due to its high cover value, abundance, and dominance in *capões* forests^38^. The botanical identification was conducted by Prof. Dr. Geraldo Alves Damasceno Junior, and the corresponding specimen was deposited in the CGMS Herbarium at the Federal University of Mato Grosso do Sul, bearing the identifier 1068446.

To enhance heterogeneity, samples were collected from 11 diverse *capões* forests in the Abrobal subregion of Pantanal. In May 2021, we collected 47 bark samples from the main stem at 1.30 m above the ground. The inclusion criteria for our study were based on a minimum diameter at breast height (DBH) of 4 cm or greater. Additionally, we consider that the diameter growth rate is directly correlated with the age of the plants. Therefore, specimens with higher DBH values were deemed mature compared to those with lower values. The samples were stored in a paper bag and left to dry at room temperature for 72 h. Afterward, they were ground in a conventional mill and the particle size was standardized using a Bead Ruptor (MM400, Retsch GmbH, Germany) for 8 min at 25 Hz.

Based on the observed patterns of fire history, the study categorized four groups: The first group, named No fire (n = 14), comprised individuals sampled in *capões* forests where the last fire had occurred in 2009. The second group, Fire 19 (n = 10), consisted of individuals sampled in *capões* forests which had experienced fires in 2019. The third group, Fire 20 (n = 13), included individuals collected from *capões* forests that had fires in 2020. The fourth and final group, APD 20 (n = 10), consisted of individuals whose aerial parts had died during the 2020 fire event. The severity of fire incidents varies greatly and is mainly distinguished by the intensity of the flames. The megafire in 2020 showcased this aspect, with the destruction of trees and vegetation being a significant feature. It is important to note that this event had a wide range of conditions. To accurately capture this diversity, we meticulously collected samples from each affected area, providing a more comprehensive understanding of the varying levels of fire intensity.

### Extraction of samples

The powdered samples (20 mg) were extracted with 2 ml of methanol:deionized water (7:3 v/v) for 10 min on an ultrasound bath (Cristofoli, 42 kHz, 2.5 l) and, subsequently, the samples were centrifuged for 5 min and the methanolic extract was obtained that was applied for the determination of total phenolic and tannin contents. From this sample, we separated 1 ml, added 10 mg of hide powder (SIGMA), and placed the product in a shaker for 60 min, followed by centrifugation for 5 min.

We followed the method of Herald et al.^39^ with modifications to evaluate the total phenolic content (TPC) and total tannin content (TTC). In a 96-well plate, we added 50 µl of deionized water to all wells, followed by 50 µl of methanolic extract (phenolic and/or tannin extract), except in the blank reagent sample wells. Using serial dilutions, we performed five different concentrations, ranging from 75 to 4.7 µl ml^−1^, obtained by transferring 75 µl of the mixture from the first well to the next. We then added 175 µL of deionized water to the control wells. Subsequently, 50 µl of *Folin-Ciocalteu* reagent diluted in deionized water (1:1 v/v) were added. After 6 min, we added 100 µl of sodium carbonate aqueous solution (Na_2_CO_3_) 75 g l^−1^, except in the wells of the control. The plate was incubated in the dark at room temperature for 90 min, and the absorbance at 765 nm was measured with a spectrophotometric microplate reader (SpectraMax®Plus384, Molecular Devices, Sunnyvale, CA, USA). We also did the blank well, which was prepared only at 50% methanol and 175 µL of deionized water, using the same method described above. Examining the blank, reagent, and control wells is to obtain reference measurements and assess potential impurities, thereby validating the analysis. The absorbance of the reagent blank is subtracted from the absorbance of the sample solution to eliminate the error. The gallic acid aqueous solution (1 mg ml^−1^) was utilized as a standard at a concentration range of 2–125 µg ml^−1^ in order to create a calibration curve (average R^2^ = 0.9989) and the concentrations of phenolic and tannin were expressed in mg g^-1^ of gallic acid equivalent.

### Statistics analysis from total phenolic and tannin contents

One-way ANOVA analyses were conducted to determine if there were differences in concentrations of TPC (total phenolic compounds) and TTC (total tannin compounds) among the studied groups. Tukey's post hoc comparisons were performed. Additionally, a generalized linear model (GLM) was used to assess the influence of DBH on TPC and TTC, and group differences were examined. All analyzes were performed on the R platform^40^. The rstatix packages were used to obtain ANOVA and Tukey's post hoc tests. The distribution of data was verified using the fitdistrplus package, while the GLM function with the Gaussian family was employed to evaluate models and interactions. The ggplot2 and vis-a-vis packages generated graphical representations for each analysis. Significance was considered at a threshold of p < 0.05.

### Thermogravimetric analyses

We used pulverized plant and dried methanol extract to analyze the species' thermal resistance and behavior of secondary metabolites under high temperatures. The extracts were obtained by the powder samples (40 mg) extracted in methanol and ultrapure water 7:3 (v/v) in an ultrasonic bath for 10 min, followed by centrifugation for 5 min. Finally, the supernatant was collected and left to dry at room temperature for 36 h. To evaluate the thermal behavior, we obtained the TGA/DTG curves by a TGA-Q50 instrument (TA Instruments, New Castle, DE, USA) and added approximately 7–9 mg of samples in a platinum crucible. The analysis was performed at a heating rate of 10 °C min^−1^ from room temperature to 900 °C under a synthetic air atmosphere with a purge flow rate of 60 ml min^−1^ in the oven and 40 ml min^−1^ of nitrogen atmosphere on balance.

### HPLC–DAD–ESI–MS/MS analyses

We extracted 40 mg of powdered plant material in methanol and ultrapure water 7:3 (v/v) with 1% formic acid. Then, these materials were extracted in an ultrasonic bath for 10 min and centrifuged for 5 min. The supernatant was filtered by syringe filters (Millex PTFE 0.22 µm, Millipore) for further analysis.

For these analyses, a Shimadzu LC-20AD HPLC chromatograph coupled to a diode array detector and a mass spectrometer ESI-QTOF MicrOTOF-Q III, Bruker Daltonics, Billerica, MA, USA) were used. The analyses were performed by monitoring the UV wavelength between 240 and 800 nm and mass spectrometer operating in negative ion mode. The chromatographic column was a Kinetex C-18 (2.6 µm, 150 × 2.1 mm, Phenomenex). The mobile phase consisted of ultrapure water (solvent A) and acetonitrile (solvent B), both added formic acid 0.1% (v/v). The gradient elution profile was the following: 0–2 min, 3% B (isocratic); 2–25 min, 3–25% B (linear gradient); 25–40 min, 25–80% B (linear gradient); 40–43 min, 80% B (isocratic); 43–44 min, 80–3% B (linear gradient), and added the time to equilibrate the chromatographic column and system (5 min). The flow rate was 0.3 ml min^−1^ and the injection volume was 4 µl for each sample. The oven temperature was 50 °C during the analyses and all samples were randomized for the injection of the system. The quality control (QC) sample consisted of a pool produced by an aliquot of 2 mg of each sample (powdered plant material) and extracted with the same method applied for the samples. The QC was injected after every 6 samples.

Data alignment was performed by Metalign software^41^ and entries were reduced by Msclust^42^. We evaluated the reproducibility of the equipment by comparing replicates and the QC samples. We also evaluated the variations of ions between the groups and the pattern of metabolites by Partial Least-Squares Discriminant Analysis (PLS-DA) and Volcano plot, respectively, using the Metaboanalyst 5.0 platform^43^. For these analyses, data were log-transformed and autoscaled. The chemical constituents from the samples were annotated based on UV, accurate mass, MS/MS data and compared to data reported in the literature.

## Results

When interpreting the results, it is essential to consider the time between the fire histories in the study area. In 2009, all the *capões* forests in the region were burned, indicating a significant fire event. However, in 2019 and 2020, the fire incidents were more localized, affecting different *capões* forests. That allowed us to observe and compare the impact of fire on the chemical composition of *R. elaeocarpum* for a minimum of two years.

### Total phenolic and tannin contents

**Figure 1:**
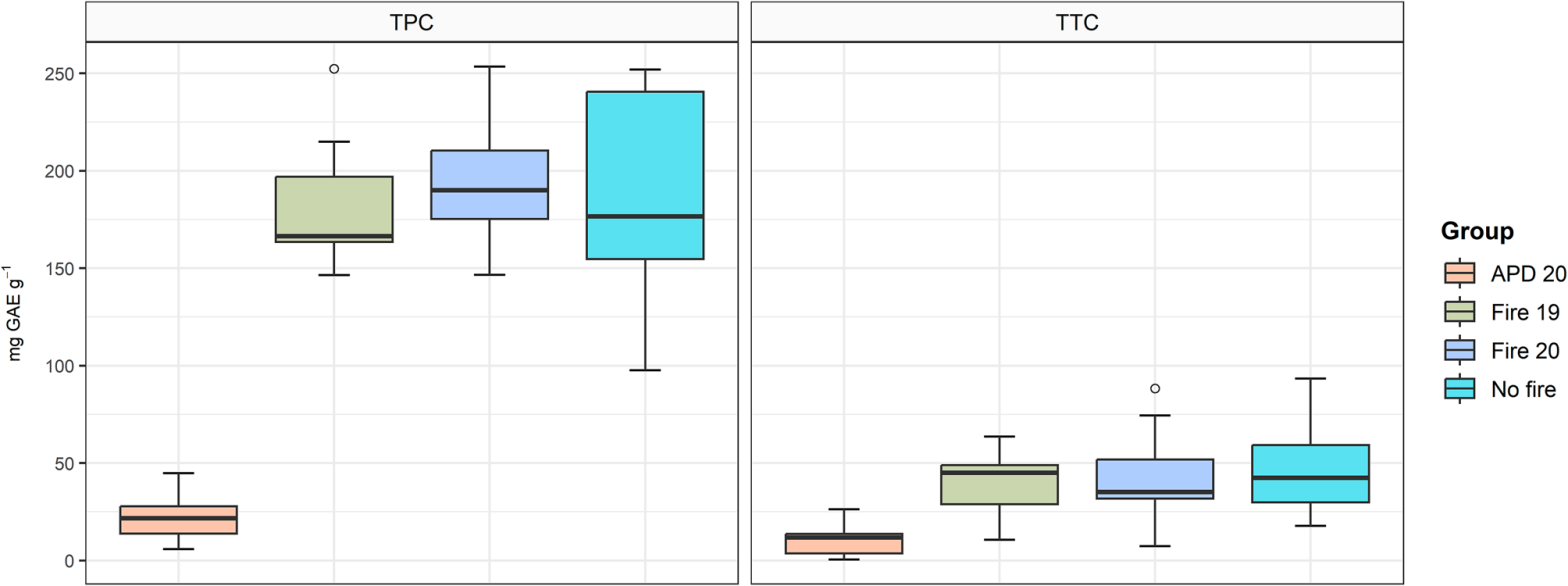
Box plot representing Total Phenolic Content (TPC) and Total Tannin Content (TTC), expressed as mg GAE g^−1^. According to Tukey's post hoc (p < 0.05), mean values with an asterisk indicate a significant difference.

We recorded high contents of phenolics and tannins from the samples, independent of fire occurrence, on *R. elaeocarpum.* The mean total phenolic content (TPC) and total tannin content (TTC) of the bark samples from individuals were expressed in mg gallic acid equivalent (GAE) g^−1^ of phenolic extract and tannin, respectively. The results obtained in the Fire 19 and Fire 20 groups were similar to those of the No fire group. The mean TPC and TTC values for individuals in the No fire group were 185.91 ± 54.21 and 47.94 ± 23.62, while the Fire 19 group were 181.47 ± 32.62 and 39.97 ± 15.39. For individuals in the Fire 20 group, the mean TPC and TTC values were 192.84 ± 30 and 42.25 ± 23.36. However, individuals in the APD 20 group showed significantly lower TPC (22.08 ± 11.73) and TTC (10.41 ± 8.27) concentrations (Fig. 1). In other words, the individuals whose aerial parts died during the 2020 wildfire experienced a substantial reduction in their phenolic metabolite content.

**Figure 2:**
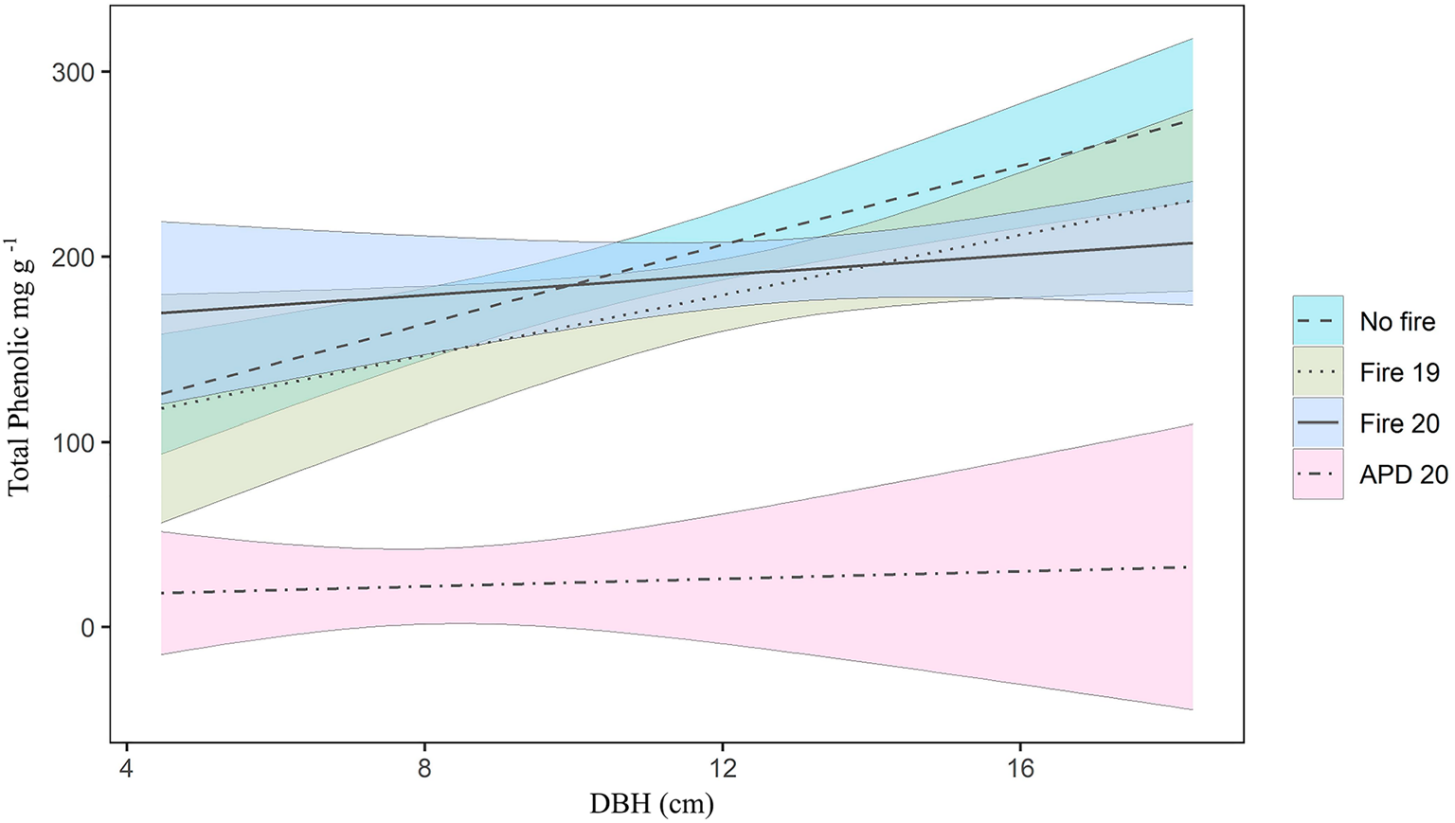
Generalized linear model (GLM) for the concentration of total phenolic in individuals of *R. elaeocarpum* in *capões* forests of Pantanal wetlands, according to fire history and DBH value (p = 0.04; Pseudo R^2^ = 0.85). The groups are represented by dashed line = No fire; dotted line = Fire 19; continuous line = Fire 20; dotdash line = APD 20. Color areas = confidence interval 95%.

Through the General Linear Models (GLM), we observed that the contents of total phenolic and tannins differed only for the *APD 20* group (Fig. 2). Furthermore, we observed an increase in the concentration of phenolic compounds in relation to DBH (Fig. 2), indicating more significant accumulation of compounds, such as gallocatechin and epicatechin-3-O-hexoside (see Figure in Supplementary Material S2), in older trees. For the low molecular weight phenolics, we observed that the No Fire and Fire 19 groups (p = 0.02 and 0.01) had a higher concentration of compounds as the DBH increased; however, there was no statistical separation between the groups (see Figure in Supplementary Material S1). On the other hand, the concentration of tannins did not present a significant difference between the groups and the relationship between the accumulation of compounds and DBH (see Figure in Supplementary Material S1).

### Annotation of the constituents from samples

**Figure 3:**
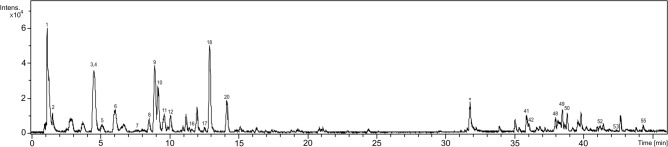
Chromatographic profile of the hydromethanolic extract from *R. elaeocarpum* by HPLC–MS/MS obtained by negative ion mode.

After processing the data from the metabolomics analysis, we detected 55 peaks and annotated 23 of the totals. The main class of metabolites observed in the study was proanthocyanidins, found as dimers in *R. elaeocarpum* (Fig. 3, Table 1). Furthermore, we highlight peaks **41–42** and **49–50**, which were putatively annotated as derivatives of karwinaphthopyranones, dimeric anthracenones.

**Table 1:** Compounds of *Rhamnidium elaeocarpum* extract by HPLC–DAD–MS/MS.

Peak	RT (min)	UV (nm)	MF	[M−H]^−^	MS/MS	Compound	References
1	1.2	–	C_12_H_22_O_11_	341.1094		Di-*O*-hexoside	
2	1.5	–	C_6_H_8_O_7_	191.0197	111	Citric acid	^44^
3	4.5	270	C_30_H_26_O_14_	609.1250	547, 474, 305	Prodelphinidin dimer type B	^45^
4	4.5	270	C_15_H_14_O_7_	305.0667	261, 219	Gallocatechin	^46,47^
5	5.2	276	C_36_H_36_O_18_	755.1829	–	Flavonol derivative	
6	6.2	275	C_30_H_26_O_13_	593.1301	423, 305	Procyanidin-prodelphinidin dimer B type	^48^
7	7.9	270	C_19_H_26_O_13_	461.1307		NI	
8	8.7	280	C_36_H_36_O_17_	739.1880	435, 273	Procyanidin dimer type B hexoside isomer	^49^
9	9.0	272	C_21_H_24_O_11_	451.1246	313, 166 161	Epicatechin-3-O-hexoside isomer	^49^
10	9.3	276	C_15_H_14_O_7_	305.0667	261, 219	Epigallocatechin	^47^
11	9.7	277	C_36_H_36_O_17_	739.1880	569, 407	Procyanidin dimer type B hexoside isomer	^49^
12	10.2	280	C_30_H_26_O_13_	593.1301	423, 305	Procyanidin-prodelphinidin dimer type B	^48^
13	11.1	280	C_51_H_48_O_22_	1011.2564	–	NI	
14	11.3	280	C_13_H_24_O_9_	323.1348	298, 165	NI	
15	11.5	278	C_59_H_62_O_33_	649.1592*	–	NI	
16	11.6	278	C_36_H_36_O_16_	723.1931	553, 407, 255	Procyanidin type B deoxyhexoside	^50,51^
17	12.6	278	C_30_H_26_O_12_	577.1351	407, 289	Procyanidin dimer type B	^49^
18	13.0	279	C_21_H_24_O_10_	435.1310	289, 271, 203	Epicatechin deoxyhesoxide	
19	13.2	279	C_21_H_22_O_11_	449.1089	–	NI	
20	14.1	279	C_16_H_20_O_10_	371.0984	249, 189	NI	
21	15.2	279	C_16_H_20_O_11_	387.0933	–	NI	
22	16.0	280	C_27_H_32_O_16_	611.1618	–	NI	
23	16.4	278	C_17_H_22_O_10_	385.1140	–	NI	
24	16.7	278	C_34_H_44_O_21_	787.2324	–	NI	
25	17.0	282	C_23_H_39_O_13_	523.2396	–	NI	
26	17.5	278	C_27_H_34_O_16_	613.1802	–	NI	
27	17.7	278	C_17_H_24_O_9_	531.0828	–	NI	
28	17.8	278	C_18_H_25_O_20_	561.0945	–	NI	
29	18.4	280	C_47_H_58_O_29_	1085.2978	–	NI	
30	18.6	278	C_28_H_40_O_13_	583.2396	–	NI	
31	19.3	279	C_29_H_30_O_16_	597.1480	–	NI	
32	19.6	278	C_41_H_50_O_24_	925.2622	–	NI	
33	20.8	280	C_45_H_54_O_28_	1041.2729	–	NI	
34	20.9	280	C_28_H_35_O_16_	627.1931	–	NI	
35	21.0	282	C_46_H_53_O_27_	1037.2780	–	NI	
36	21.1	280	C_54_H_52_O_23_	1067.2827	–	NI	
37	21.3	280	C_64_H_76_O_40_	742.1962*	–	NI	
38	22.9	282	C_48_H_56_O_27_	1063.2936	–	NI	
39	24.4	280	C_19_H_34_O_10_	421.2079	–	NI	
40	31.5	270	C_18_H_33_O_5_	329.2333	–	NI	
41	35.8	278, 429	C_30_H_26_O_8_	513.1555	495, 471, 410, 57	karwinaphthopyranone derivative	^52^
42	36.0	278, 429	C_30_H_26_O_8_	513.1555	495, 471, 410, 57	karwinaphthopyranone derivative	^52^
43	36.5		C_30_H_24_O_8_	511.1411		NI	
44	36.7		C_18_H_30_O_3_	293.2112	–	NI	
45	36.8		C_34_H_60_O_16_	723.3809	–	NI	
46	37.1		C_30_H_28_O_8_	515.1741	–	NI	
47	37.3		C_34_H_44_O_9_	595.2913	–	NI	
48	37.9	–	C_18_H_32_O_3_	295.2279	277, 171	( ±)9-hydroxy-10E,12Z-octadecadienoic acid (9-HODE)	^53^
49	38.4	268, 413	C_32_H_32_O_8_	543.2024	528, 513	karwinaphthopyranone derivative	^52^
50	38.8	268, 413	C_32_H_32_O_8_	543.2024	528, 513	karwinaphthopyranone derivative	^52^
51	40.9	–	C_37_H_16_O_3_	507.1027	–	NI	
52	41.3	–	C_18_H_30_O_2_	277.2173	–	Fatty acid	
53	42.5	–	C_18_H_32_O_2_	279.2330	–	Fatty acid	
54	43.7	–	C_16_H_32_O_2_	255.2330	–	Fatty acid	
55	44.2	–	C_18_H_34_O_2_	281.2486	–	Fatty acid	

*RT* retention time, *MF* molecular formula, *NI* not identified.

*Double charge.

**Compound 1** revealed the chemical formula C_12_H_22_O_11_, with no absorption in the UV spectra, and was defined as hexoside dimer. **Compound 2** demonstrated an ion at *m/z* 191.0197 and a fragment ion at *m/z* 111, corresponding to the loss of one CO_2_ and two water molecules [M−H-44-18-18]^−^; this fragmentation profile matches citric acid, as observed by Peng et al.^44^.

**Compounds 4** and **10**, molecular formula C_15_H_14_O_7_, showed an intense ion at *m/z* 305.0667, which yielded the fragment ion at *m/z* 219 by the loss of a unit of C_3_O_2_ [M-H-88]^-^. Although they have similar fragmentation patterns, the large discrepancy in retention time between them indicates to be isomers. According to Sakakibara et al.^47^, we have a pattern for the given characteristics, where the compound with the shortest retention time corresponds to gallocatechin, in our case, peak 4. Epigallocatechin has a longer retention time, represented by peak 10 in this study. The **compound 18**, is epicatechin *O*-deoxyhexoside, has molecular formula C_21_H_24_O_10_ and deprotonated ion at *m/z* 435.1310. We observed the product ion at *m/z* 289, yielded by loss of deoxyhexose [M–H-146]^−^, followed by the loss of a water molecule [M−H-146-18]^−^ and A ring [M−H-68]^−^.

**Compound 5**, chemical formula C_36_H_36_O_18_, reveals a UV spectrum compatible with the characteristic bands of flavonols. However, the second peak corresponding to the 330–350 nm range showed low intensity in the UV spectrum.

**Compound 3** exhibited ions *m/z* 609.1250 and **compounds 6** and **12** m*/z* 593.1301, which correspond to C_30_H_26_O_14_ and C_30_H_26_O_13_, characterizing respectively as prodelphinidin dimer type B and procyanidin-prodelphinidin dimer type B, reported by Martini et al.^49^. The peaks showed fragment ions at *m/z* 305 yielded from Quinone Methide (QM) reactions with losses of 304 and 288 *u*; thus, prodelphinidin and procyanidin units were confirmed. The fragment ions *m/z* 423 observed for **peak 6** and **12** are products of the retro-Diels–Alder (RDA) reaction [M−H-152-H_2_O]^−^. The **compounds 8** and **11**, molecular formula C_36_H_36_O_17_ and *m/z* 739.1880 were annotated as isomers of procyanidin dimer type B hexoside with different fragments. **Compound 8** showed a product ion at *m/z* 435 yielded by two consecutive RDA fissions [M−H-152–152]^−^, followed by hexose loss [M−H-162]^−^. **Metabolite 11** had a loss of 152 *u* by RDA with subsequent elimination of a water molecule [M−H-152–18]^−^, generating the ion *m/z* 569, followed by loss of hexose [M−H-162]^−^ that resulted in the fragment *m/z* 407. **Peak 16** is a derivative of the **compounds 8** and **11**, with a deoxyhexose moiety. **Peak 17**, is relative to a procyanidin dimer type B, which exhibited a deprotonated ion at *m/z* 577.1351, compatible with the molecular formula C_30_H_26_O_12_. The fragmentation pattern matches two common pathways of procyanidins; the fragment *m/z* 407 is the product of RDA fission, followed by the loss of a water molecular [M−H-152-18]^−^ and QM reaction produced fragment *m/z* 289. The fragmentation pathways of procyanidins annotated in the study are consistent with previously reported data^54–57^.

**Compounds 41–42** and **49–50** exhibited identical UV spectra with bands at approximately 278 and 429 nm, compatible with anthracenones, from the deprotonated ions at *m/z* 513.1555 and *m/z* 543.2024, and indicate the molecular formula C_30_H_26_O_8_ and C_32_H_32_O_8_ respectively. We putatively annotated them as derivatives karwinaphthopyranones, compatible with the structure identified by^52^. However, confirmation is not possible due to the lack of information on fragmentation patterns in the literature. The compounds are formed by dimeric hydroxyanthracenones, and are characteristic of genera of the Rhamnaceae family^52,58,59^.

Through the MS/MS spectrum, we annotated **compound 48**, deprotonated ion at *m/z* 295.2279 and molecular formula C_18_H_32_O_3_, as 9-HODE, classified as an oxidized derivative of linoleic acid, and based on the typical fragmentation pattern as reported by Yuan et al.^53^. The ion *m/z* 277 resulted from the loss of H_2_O, followed by cleavage between the carbon–carbon bond adjacent to the hydroxyl group, generating the main fragment at *m/z* 171. **Compounds 52***,* **53***,* **54***,* and **55** were annotated as fatty acids due to the strong interaction between the metabolites and the stationary applied here, with the chemical formulas C_18_H_30_O_2_, C_18_H_32_O_2_, and C_18_H_34_O_2_, respectively, in addition to not having UV spectra.

### Metabolomics analyses

**Figure 4:**
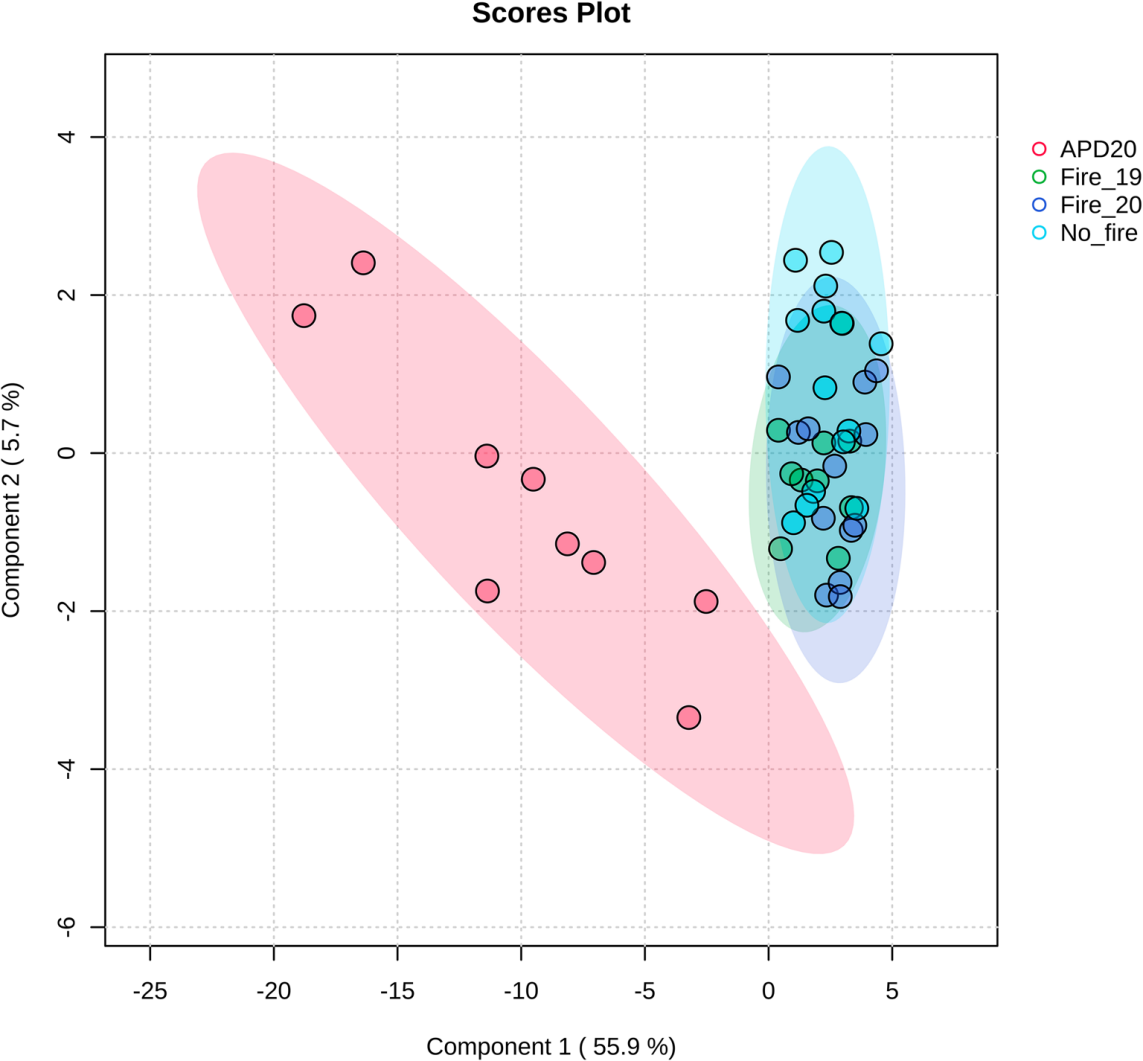
Partial least squares-discriminate analysis (PLS-DA) plot of different metabolites in *R. elaeocarpum* groups. PLS-DA score plot for APD 20 (red), Fire 19 (green), Fire 20 (purple) and No fire (blue).

Partial least squares discriminant analysis (PLS-DA), which recognizes patterns through supervised classification, showed distinctions only between the compounds of the APD 20 group and the others (Fig. 4). PLS-DA explained 61.6% of the data variability and components 1 and 2 explained 55.9 and 5.7%.

**Figure 5:**
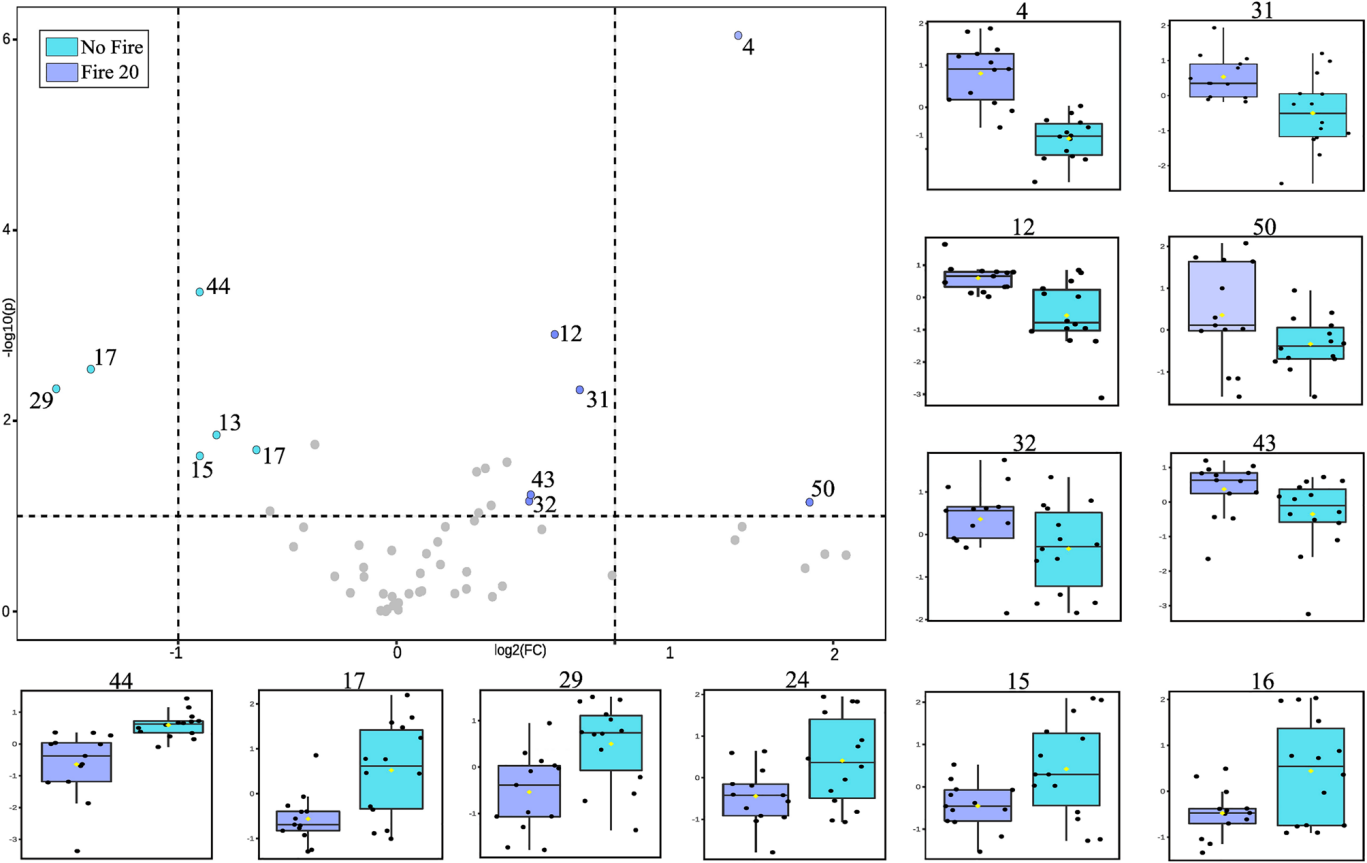
Volcano plot of metabolite concentration changes by fire history, considering the P-value threshold of 0.01 and fold change (FC) threshold of 1.5. Blue/purple circles show metabolites significantly accumulated for No Fire and Fire 20 groups; symbols in grey show unchanged metabolites. In addition, a box plot with a distribution of the intensities of the ions in *R. elaeocarpum*.

The volcano plot analysis unveiled qualitative disparities among the groups, emphasizing the concentration of metabolites in relation to fire occurrences. When comparing the No Fire and Fire 20 groups (Fig. 5), 12 compounds exhibited statistical significance and notable concentration changes. Gallocatechin and peak 29 concentrations displayed considerable variations in each respective group. Additionally, a robust association between one of the derivatives of karwinaphtapyranones, peak 50, and the fire event was observed.

**Figure 6:**
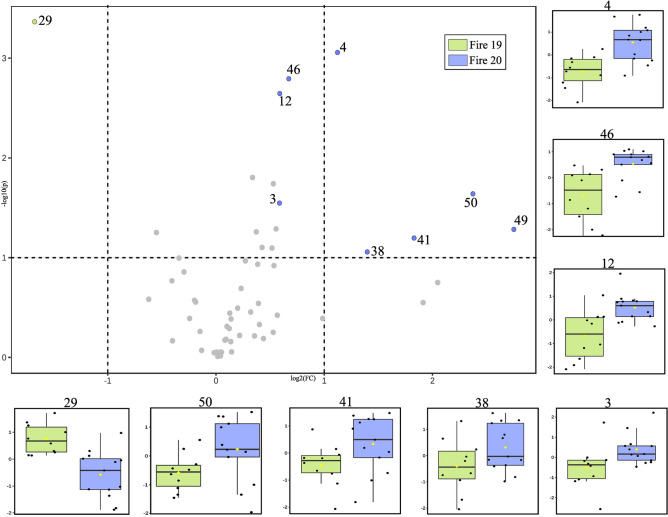
Volcano plot of metabolite concentration changes by fire history, considering P-value threshold of 0.01 and fold change (FC) threshold of 1.5. Green/purple circles show metabolites with significant concentration for Fire 19 and Fire 20 groups; symbols in grey show unchanged metabolites. In addition, box plot with an average distribution of the intensities of the ions in *R. elaeocarpum*.

Nonetheless, a comparison of the Fire 19 and Fire 20 groups (Fig. 6), which had recently experienced fire events, revealed a more pronounced correlation between karwinaphthopyranones derivatives and fire, suggesting that this compound serves as a marker for stress in *R. elaeocarpum*. Moreover, peak 29 also exhibited significant relevance in the Fire 19 group, indicating that its concentration is inherently linked to the temporal scale of the fire rather than being specific to a single event. Furthermore, the ionic intensities of secondary metabolites exhibited significant variation among individuals, irrespective of their group, indicating a high level of heterogeneity within the samples.

### Thermogravimetric behavior

**Figure 7:**
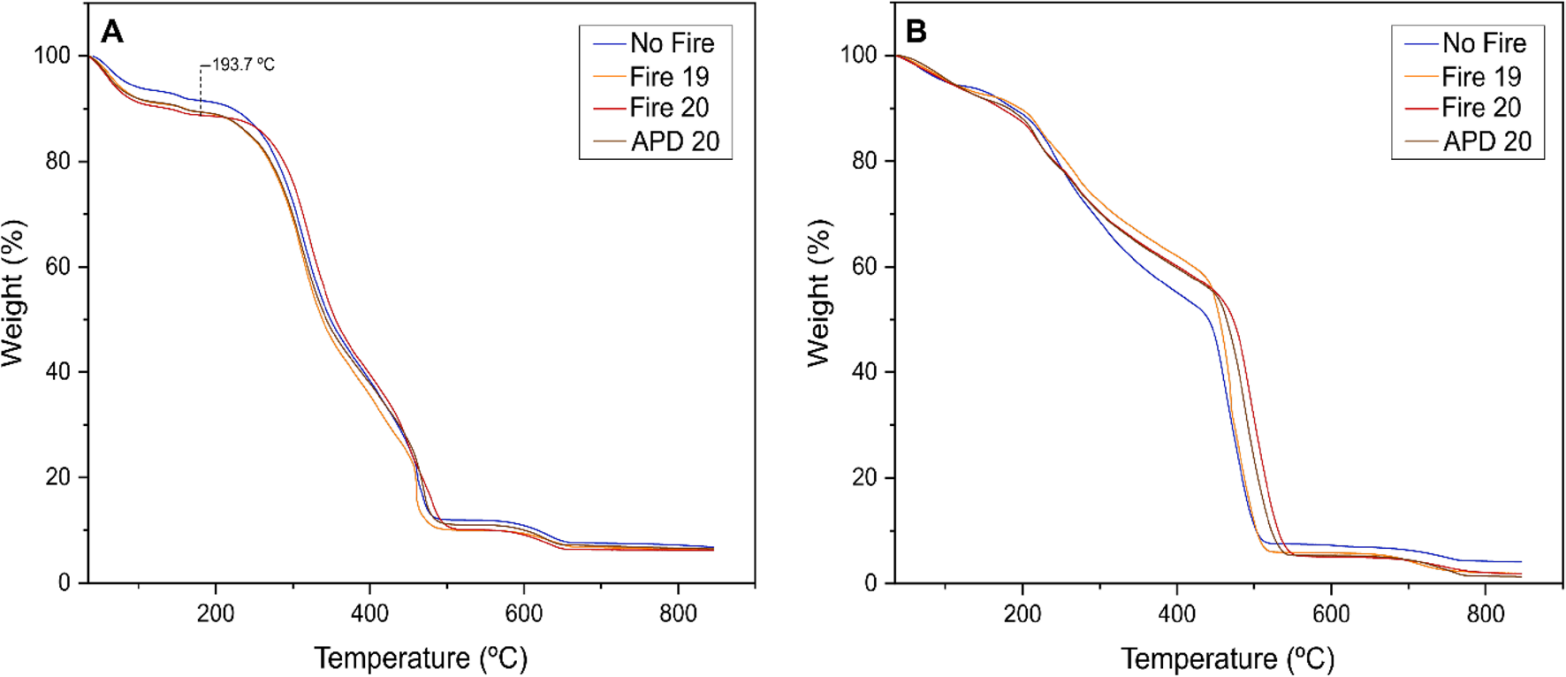
Thermogravimetric analysis (TGA) of plots of *R. elaeocarpum* from *capões* forests from Pantanal wetlands. (**A**) Thermogravimetric curves of bark powder. (**B**) Thermogravimetric curves of the dry extract. The groups are represented by blue (No fire), orange (Fire 19), red (Fire 20) and brown (APD 20).

The fire groups exhibited similar combustion patterns, with an average temperature of 193.7 °C at which the thermal decomposition of barks began (Fig. 7A). The first curve indicated a loss of about 9.17% of moisture content, followed by a significant loss of biomass, with the second thermogravimetric curve resulting in an initial average loss of 48.08%, and a subsequent reduction of 31.60%. The coefficient of variation for the curves ranged from 0.03 to 0.14%. Similarly, the TGA graph of dry extracts of *R. elaeocarpum* showed two intense peaks of biomass loss in the second curve at average temperatures of 223.42 °C and 468.44 °C, indicating a clear separation (Fig. 7B).

## Discussion

Our findings show that *R. elaeocarpum* bark contains high levels of total phenolic and tannin in all sampling groups, except APD 20 (as seen in Fig. 2). This indicates that the species' overall phenolic metabolism remains unaffected by recent fire events. It is worth noting that the region has experienced low-intensity fire for a considerable time, suggesting that the plant has developed a biochemical tolerance to the physiological stress caused by fire. Although, Da Silva et al.^21^ documented elevated levels of phenolics in thermotolerant species from the Pantanal, our study goes beyond these findings by illustrating that this trait is not solely associated with fire. Moreover, we emphasize the significance of our results in the realm of fire ecology and the conservation of fire-affected ecosystems, underscoring the necessity of accounting for additional environmental disturbances when examining the phenolic compositions of thermotolerant species in the Pantanal.

In 2020, during this study, the Brazilian Pantanal experienced its most severe and prolonged fire event, attributed to an extended period of intense drought and high temperatures^2^ The detrimental impacts of the fire were observed in the *R. elaeocarpum* population, leading to topkill in specimens of the APD 20 group. The loss of aboveground structures due to intense fires can be attributed to various factors, such as cambium necrosis^60,61^, xylem damage^62,63^, and phloem and crown mortality^64^. The intense damage inflicted upon the APD 20 group resulted in bark decomposition, which in turn contributed to the observed leaching of biomass, explaining the significant difference in total phenolic levels compared to the other groups. When leached or released from decomposing plant residues, phenolics can play a significant ecological role through allelopathic action by affecting other organisms' germination, development, and reproduction. Therefore, due to their effects, phenolics are considered one of the most important and common plant allelochemical classes^65,66^.

Chemical variation plays a crucial role in promoting biodiversity, enabling species to establish and persist in dynamic environments^67^. These variations within species regarding their secondary metabolites are attributed mainly to phenotypic plasticity, as population genotypes adaptively respond to the local abiotic and biotic environment in which they thrive^68^. The high standard deviation values of TPC and TTC concentrations in *R. elaeocarpum* (Fig. 1) indicate significant intrapopulation variability in response to fire, contributing to adaptation to changing environmental conditions^25,69^. The observed differences in the species' population align with previous studies^25,67,68^.

The analysis of metabolism revealed that there was a higher accumulation of proanthocyanidin oligomers in *R. elaeocarpum*, and no significant differences were observed between the groups (see Fig. 4 and Table 1). This finding suggests that the synthesis of these compounds has not been triggered by fire within the past six months. The biochemical response of the species may be an exaptation by environmental factors other than fire, such as high levels of radiation, greater daily and seasonal temperature range, and drought^3,25^. Previous studies have reported an increase in the production of phenolic compounds in response to changes in UV radiation, which is associated with the stimulation of antioxidant defenses^70,71^. Furthermore, proanthocyanidins induction in response to herbivory has been observed in several species of woody trees^72–74^. Hence, while the synthesis of proanthocyanidin oligomers in *R. elaeocarpum* may not be a directly adaptive trait associated with fire, its presence could still protect against fire-induced damage and post-fire conditions.

Moreover, proanthocyanidins and flavan-3-ol monomers play critical ecological functions in environments with recurrent fires due to their excellent antioxidant capacity, resulting from their numerous hydroxyl groups^75,76^. Thus, we can assume that the prior presence of these compounds acted as a protective barrier against heat transfer during fires^22–24^.

The volcano graphs (Figs. 5 and 6) indicate that only individuals in the Fire 20 group exhibited noticeable changes in their chemical profile, leading to the accumulation of several compounds, such as gallocatechin. The antioxidant properties of this compound raise the hypothesis that the metabolite acts in post-fire protection, possibly playing a role in reducing oxidative stress and cell recovery. Moreover, the accumulation of this metabolite may be related to its function in the defense against pathogenic microorganisms that can proliferate in post-fire conditions, taking advantage of the reduction of competition and the natural defenses of the affected plants^77^. Results suggest that *R. elaeocarpum* can maintain metabolic balance even under environmental disturbances without significant quantitative changes (Fig. 4).

Additionally, the accumulation of compounds increased with age, particularly in individuals with higher DBH (Fig. 2). Altered metabolic responses are crucial for the survival and resilience of species. Organisms must experience changes in metabolic pathways and an increased accumulation of certain compounds to adapt better to their new circumstances. This adaptation gives them the ability to thrive and survive in post-fire environments.

The effects of fire and post-fire conditions have significant implications for plant species and ecological communities. Fire triggers ecosystem succession, resulting in different responses within the same environment^78^. It is common for plant diversity to increase shortly after the event as nutrients are released, light becomes available, and competition is temporarily reduced, promoting the fitness of grasses^79,80^. However, as the canopy recovers, there is a decrease in the abundance of small species^79,81^. Nevertheless, the prolonged and intensified fire regime is inducing changes in the natural dynamics of ecosystems, compromising the resilience of communities and leading to the expansion of open landscapes with limited resources^2^. de Oliveira et al.^8^ have already highlighted the necessity of analyzing the synergistic effects of environmental forces in the Pantanal plain to preserve ecosystem integrity and ensure long-term sustainability.

The passage of fire also led to a high incidence of karwinaphthopyranone derivatives, as observed in Figs. 5 and 6. These metabolites have been extensively studied in *Karwinskia*, a genus closely related to *Rhamnidium*^82,83^. Some hydroxyanthracenones, have been shown to exhibit antimicrobial and antifungal activities^84,85^ and toxic properties^52,58,86,87^. This finding serves as a warning to the Pantanal population who have been using *R. elaeocarpum* barks for food and therapeutic purposes^29,30,88^, since the effects of long-term effects on the organism are still unknown.

Based on the thermogravimetric analysis, *R. elaeocarpum* maintained its stability up to an average temperature of 193.7 °C (Fig. 7A), classifying the species as thermotolerant according to the criteria established by Da Silva et al.^21^. This result suggests an excellent resistance to the local fire regime, typically involving low-intensity fires. We believe this adaptation is linked to the high concentrations of proanthocyanidins in the bark. The TGA graph of the dry extract (Fig. 7B) clearly shows that the second curve splits and indicates two significant biomass losses, a characteristic behavior of condensed tannins^89^. We hypothesize that these losses were mainly due to polymer breakdown and subsequent degradation of proanthocyanidin monomers.

## Conclusion

Our results indicate that fire stress and post-fire conditions act as modulators in the plant community, altering the metabolic balance with a lasting qualitative impact for at least 2 years. The high concentration of phenolic in the bark of *R. elaeocarpum* contributes to its thermotolerance, protecting against seasonal damage and aiding in resilience in post-fire environments. We also observed a tendency for metabolite accumulation in older individuals, indicating the species' chemical defense against successive environmental stresses. In addition, the metabolomics results highlighted antioxidant compounds such as proanthocyanidins, which protect against damage by reactive oxygen species in metabolic processes. However, the responses observed after different fire histories cannot be attributed solely to fire but also to a combination of drought, high solar incidence, and common pathogenic responses in post-fire environments, describing an adaptive trait known as exaptation. Our findings also indicate that conservation and educational actions are essential for local communities that use *R. elaeocarpum* for medicinal purposes due to toxic compounds in the plant, such as karwinaphtopyranone derivatives. Promoting awareness of the risks associated with indiscriminate plant use without proper guidance. To deepen the understanding of the adaptive strategies employed by plants, future studies should focus on investigating the influence of different fire intensities on the chemical composition of *R. elaeocarpum* and other plant species, contributing to knowledge about the diversity of chemical compounds and their ecological roles in the wetland ecosystem.

## Supplementary Information

Supplementary Figures.

## Data Availability

Untargeted metabolomics data have been deposited to Science Data Bank repository. The registered database DOI is https://doi.org/10.57760/sciencedb.08078.
