# Supplementary material for: Unraveling the adaptive chemical traits of Rhamnidium elaeocarpum Reissek in response to fire in pantanal wetlands
Source: Sci Rep. 2023 Jul 22;13:11860. doi: 10.1038/s41598-023-38725-2 (PMC10363117; doi:10.1038/s41598-023-38725-2)
Supplement: Supplementary file 1 — Supplementary Figures. [file 41598_2023_38725_MOESM1_ESM.pdf]

Supplementary Material for Unraveling the adaptive chemical traits of *Rhamnidium elaeocarpum* Reissek in response to fire in Pantanal wetlands

C. S. Siqueira, PPGBV-UFMS and V. S. dos Santos, FACFAN-UFMS and C. A. Carollo*, FACFAN-UFMS, and G. A. Damasceno-Junior, INBIO-UFMS

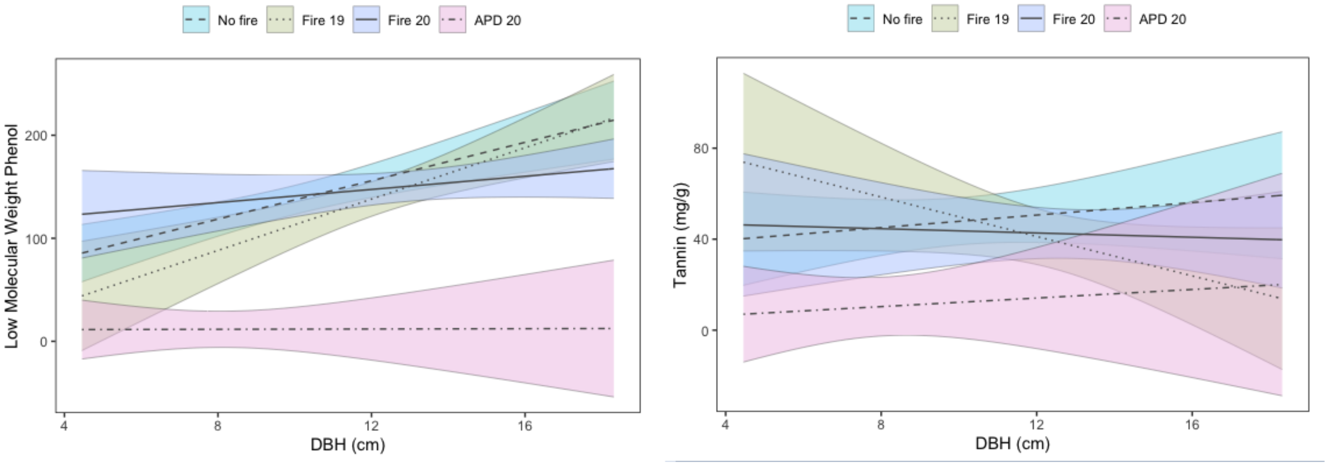

S-1: Generalized linear model (GLM) for the concentration of secondary compounds in individuals of *Rhamnidium elaeocarpum* in *capões* forests of Pantanal Abobral, according to fire history and DBH. **A.** Compounds correponding to Low Molecular Weight Phenol value ($p = 0.02$; Pseudo $R^2 = 0.84$) and **B.** Corresponding to Tannin value ($p= 0.1$;Pseudo $R^2= 0.40$). The groups are represented by dashed line = No fire; dotted line = Fire 19; continuous line = Fire 20; dotdash line = APD 20. Color areas= confidence interval 95%.

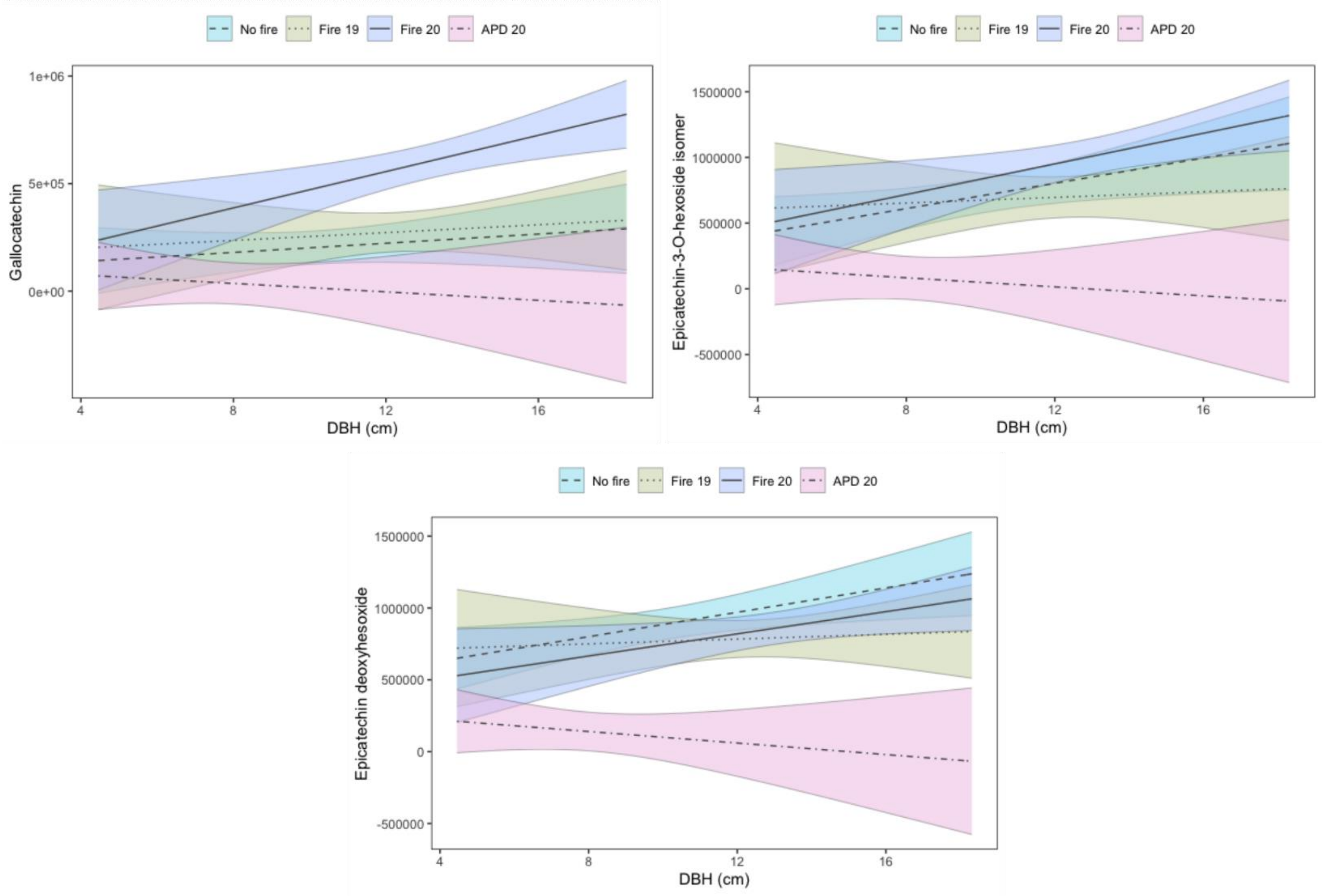

S-2: Generalized linear model (GLM) for the concentration of secondary compounds in individuals of *Rhamnidium elaeocarpum* in *capões* forests of Pantanal Abobral, according to fire history and DBH. **A.** Compounds correponding to Gallocatechin value (p = 0.2; Pseudo R^2 = 0.72), **B.** Corresponding to Epicatechin-3-O-hexoside value (p=0.04; Pseudo R^2= 0.69) and **C.** Corresponding to Epicatechin deoxyhesoxide value (p= 0.04; Pseudo R^2= 0.72). The groups are represented by dashed line = No fire; dotted line = Fire 19; continuous line = Fire 20; dotdash line = APD 20. Color areas= confidence interval 95%.

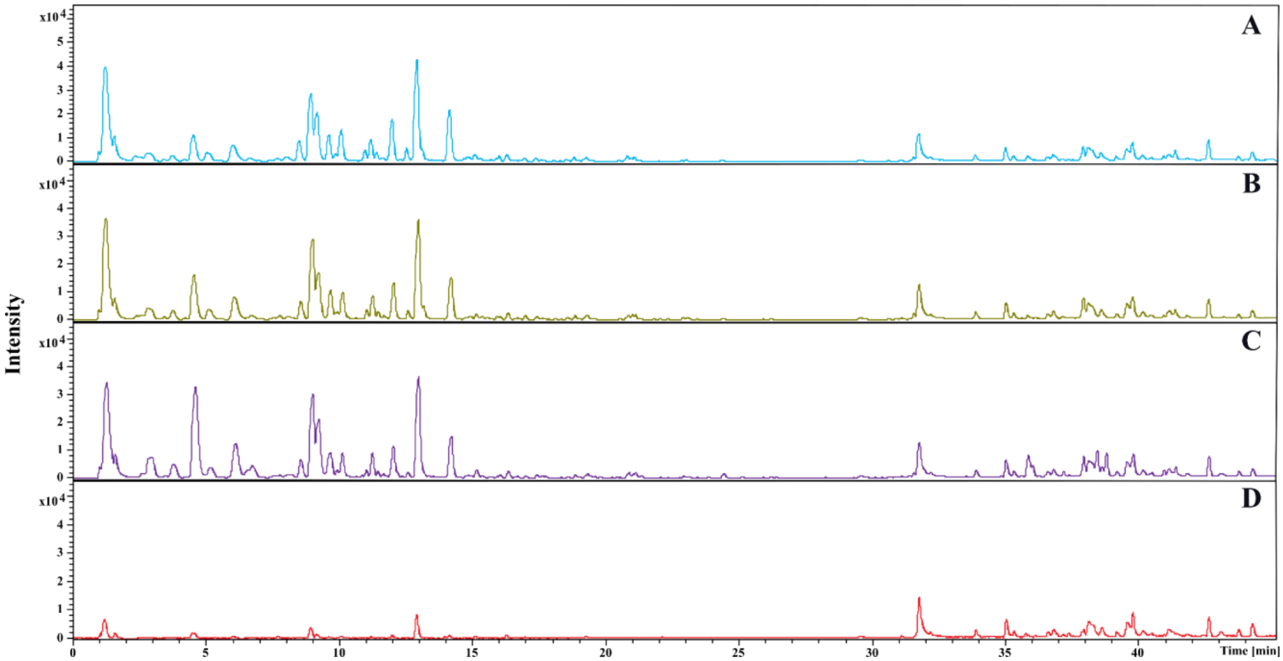

S-3: Comparison of spectrum profiles of the four sample groups. The mass spectra are represented by A. No fire (blue), B. Fire 19 (green), C. Fire 20 (purple), D. APD 20 (red).
